# Comparative population genomics analysis for chicken body sizes using genome-wide single nucleotide polymorphisms

**DOI:** 10.5713/ab.24.0347

**Published:** 2024-10-28

**Authors:** Sensen Yan, Chaoqun Gao, Kaiyuan Tian, Chengpeng Xiao, Junlai Shi, Xintao Jia, Kejun Wang, Guirong Sun, Donghua Li, Wenting Li, Xiangtao Kang

**Affiliations:** 1 College of Animal Science and Technology, Henan Agricultural University, Zhengzhou 450046, China; 2 The Shennong Laboratory, Zhengzhou 450046, China

**Keywords:** Body Size, Genome-Wide Single Nucleotide Polymorphism (SNP)s, Population Structure, Runs of Homozygosity (ROH) Islands, Selection Signatures

## Abstract

**Objective:**

This study aims to investigate the selection history, genome regions, and candidate genes associated with different chicken body sizes, thereby providing insights into the genetic basis of complex economic traits such as chicken body size and growth.

**Methods:**

In this study, a total of 217 individuals from eight breeds were selected. According to body size, they were divided into two groups: large chickens and bantam chickens, with four breeds in each group. Firstly, we investigate population structure by principal component analysis (PCA), phylogenetic tree, and ancestry component analysis. Next, we recognize runs of homozygosity (ROH) islands through calculating ROH. Finally, we carry out selection signatures analysis utilizing population differentiation index and nucleic acid diversity.

**Results:**

The population structure analysis show that large and bantam chickens are clearly separated. Large chickens are clustered together, the bantam chickens are relatively dispersed. The results of ROH island analysis show that 48 and 56 ROH islands were identified in large and bantam chickens respectively. Among the interesting ROH islands, a total of eight candidate genes were identified. In selection signatures analysis, a total of 322 selected genes were annotated in large chickens, such as *POU1F1*, *BMP10*, enrichment in 16 gene ontology (GO) terms. In bantam chickens, a total of 447 selected genes were annotated, such as *IGF1*, *GRB10*, enrichment in 20 GO terms and 2 Kyoto encyclopedia of genes and genomes pathways. The haplotype analysis results show that *GRB10* has differences in chickens of different body sizes.

**Conclusion:**

By population structure, ROH islands, and selection signatures analysis, we have identified multiple genes associated with chicken body size, growth, and development (such as *BMP10*, *IGF1*, *GRB10*, etc). This provides a theoretical reference for the subsequent development of molecular markers for chicken body size and the analysis of the genetic mechanism of chicken body size.

## INTRODUCTION

Livestock serves as excellent biological models widely utilized in developmental biology and phenotypic evolution research. Over years of improvement and selective breeding, animals have been developed into different breeds, exhibiting significant variations in morphology, physiology, behavior, and adaptation [1–3]. For the body size of livestock, there have been many previous studies. For example, some studies have analyzed and revealed genes related to horse body size by using the method of comparative population genomics [4]. Moreover, there are also other studies have also found new loci that may control the body size variation of chickens by using population genomics analysis [5]. Remarkable changes in body size can be observed in livestock, far surpassing that of their wild ancestors. There are also studies showing that deletions in the promoter region of the chicken *IGF2BP1* are associated with the body size [6]. Chickens exhibit the most remarkable phenotypic variations among poultry species [1]. In terms of body size, there is an astonishing disparity between large broiler chickens or fighting cocks (over 5 kg) and bantam chickens (around 0.5 kg), with a difference of over tenfold. For instance, the weight of cochins and bantams is about 5 kg and 0.5 kg, respectively. As the most widely distributed and abundantly raised farm animal globally, chickens are also extensively used in genetic and medical research. Numerous previous reports have analyzed genetic variation in specific traits, particularly in body size, has been extensively studied and characterized, because of their significance in both research and breeding [2,3,7].

The extensive phenotypic diversity among different chicken breeds and their complex population history have posed challenges in studying the genetic mechanisms underlying variations in chicken body size [8]. However, with the continuous advancements in genome sequencing technologies, unraveling the genetic basis behind complex traits in livestock has become increasingly feasible. For instance, cold adaptation in high-latitude Chinese pigs [9] and adaptation to starch-rich diets in dogs [10] have been successfully elucidated. Domestication and selective breeding have led to domestic chickens becoming one of the most diverse animals in terms of phenotypic variation. Body size, as an economically important trait, plays a crucial role in the development of human society, and has received long-term attention in chicken breeding. Selective breeding aimed at feed efficiency has played a pivotal role in the miniaturization of chickens, both in egg-laying and ornamental breeds. For instance, the grain-saving small-sized laying hens independently cultivated in China (Nongda No. 3 laying hens) consume approximately 12 kg less feed by the age of 72 weeks compared to ordinary laying hens. Additionally, the white-feathered broilers known as Guangming No. 2 can reach a remarkable 2.8 kilograms in body weight within a mere 42 days, as a crucial means of sustenance and income, breeders have made strenuous efforts to cultivate these large chickens that grow with astonishing swiftness. This is highly important for meat production. In our research, Brahma (BHxx), Cochin (COsch), Langshan (LSxx), and Orpington (ORge) are large chicken breeds, with mature males and females reaching weights of up to 4.6 kg and 3.8 kg, respectively. BHxx with its largest roosters weighing up to 8 kg and hens reaching 6 kg. Antwerp Bearded (ABwa), Booted Bantam (FZxx), Barbue du Grubbe (GBxx), and Sebright Bantam (SBxx) are world-famous bantam chicken breeds celebrated for their diminutive body size. These makes them excellent models for studying the body size and growth development of chickens.

The main aim of this study is to investigate the genes related to chicken body size; body size has long been a crucial consideration in breeding. Conducted at the whole-genome level, this research is designed to provide a more thorough understanding of the genetic foundation associated with chicken body size, thereby facilitating the development and progress of breeding work.

## MATERIALS AND METHODS

### Data and sample collection

The data for this study was obtained from the Synergistic Plant and Animal (SYNBREED) project (www.synbreed.tum.de). The research included a total of 217 individuals from eight different chicken breeds. Among these, there were 101 individuals from four large chicken breeds (BHxx, Cosch, LSxx, Orge) and 116 individuals from four bantam chicken breeds (Abwa, FZxx, GBxx, SBxx), detailed information can be found in (Table 1).

### Genotyping

The genotyping of DNA samples was conducted using the Affymetrix Axiom TM 600K Whole Genome Chicken Genotyping Array, which comprises over 580,000 single nucleotide polymorphism (SNP)s [11]. The genotyping array was annotated using the Gallus_gallus-5.0 reference genome [12], resulting in annotations for a total of 579,621 SNPs.

### Data filtering

Firstly, 27,416 SNPs that were duplicates, ambiguous annotations, or were located on the sex chromosomes were excluded from the SNP array. Subsequently, the remaining 552,205 SNPs underwent additional quality control using PLINK 1.9 [13]. The quality control criteria included a genotyping missing rate<0.01 and a minor allele frequency>0.05, a total of 425,164 SNPs were retained. For the subsequent analysis of population structure, an additional linkage disequilibrium filtering is necessary. The filtering parameters, including window size, step size, and r2, are set as “50, 5, 0.2”. Finally, 45,294 SNPs were retained for the population structure analysis.

### Data analysis

#### Population structure analysis

PLINK 1.9 was used to conduct principal component analysis (PCA) with genome-wide SNPs. Utilize the VCF2Dis v.1.42 ( https://github.com/BGI-shenzhen/VCF2Dis ) to calculate the genetic distance matrix, this matrix quantifies the genetic differences or similarities between individuals or populations, the neighbor-joining method in MEGA X [14] was used to construct a phylogenetic tree based on the genetic distance matrix. Employed the iTol website ( https://itol.embl.de/ ) to annotate and visualize the constructed phylogenetic tree. The Admixture (v1.3.0) [15] was used to analyze population structure. The optimal ancestries population number (K) within the studied populations was determined through cross-validation error rate estimation.

#### Runs of homozygosit y islands analysis

PLINK 1.9 can be employed to analyze ROH for each individual. The ROH occurrence rate is the percentage of animals within a given population that have SNPs in their ROH segments. The top 1% is selected as the region under selection, and it is visualized using a Manhattan plot in R. The detection of ROH islands sets the minimum threshold at 30% and the maximum threshold at 80%. This means that ROH must be present in at least 30% of individuals, and all ROH with occurrence rates higher than 80% will be labeled as ROH island [16].

#### Selecting signatures analysis

Used VCFtools [17] to calculate the population differentiation index (Fst) and nucleic acid diversity (π) values between populations. The sliding window was set with a window size of 50 Kb and a step size of 10 Kb. In this study, the Fst and π ratio methods were used for selection signatures analysis. The candidate genomics were determined by taking the top 5% value range as the threshold. Subsequently, gene annotation of the candidate regions was carried out. Perform haplotype analysis on the region with obvious selection signatures.

#### Annotation and enrichment analysis

To further comprehensively analyze the biological functions of the obtained candidate genes in a systematic manner, gene ontology (GO) enrichment analysis and Kyoto encyclopedia of genes and genomes (KEGG) pathway analysis were performed using the online analysis platform DAVID 6.8 [18]. The level threshold was set as p-value<0.05.

## RESULTS

### Population structure analysis

**Figure 1:**
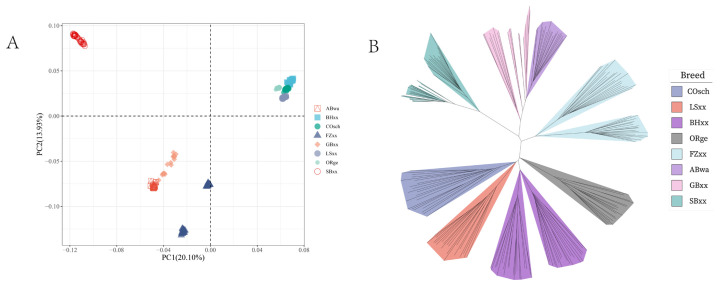
PCA and phylogenetic tree of large and bantam chickens. (A) PC1 effectively separates the large chickens from the bantam chickens, the large chickens cluster together with closer genetic distances within the group, the bantam chickens show relatively more dispersed clustering and greater genetic distances within the group. (B) Large chickens are located at the bottom, bantam chickens are at the top, and both BHxx, SBxx, and FZxx contain two subgroups. PCA, principal component analysis; PC, principal components; BHxx, Brahma; SBxx, Sebright Bantam; FZxx, Booted Bantam.

The PCA results show the contribution rates of the first two principal components: PC1 (20.10%) and PC2 (13.93%). PC1 effectively separates the large chickens from the bantam chickens, the large chickens cluster together with closer genetic distances within the group, the bantam chickens show relatively more dispersed clustering and greater genetic distances within the group. It is worth noting that ABwa and GBxx cluster together, this observation aligns well with the findings from the phylogenetic tree analysis (Figure 1). In both the phylogenetic tree and ancestry component analysis, the FZxx, SBxx and BHxx populations are observed to have two subpopulations. However, in the PCA, only the FZxx population exhibits clear separation into two subpopulations, while the SBxx and BHxx populations do not show separation.

**Figure 2:**
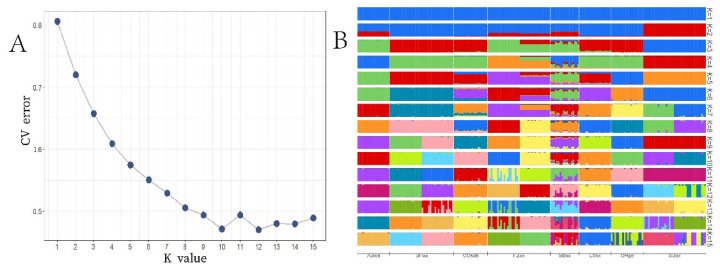
Optimal path of K values and ancestry component analysis of large and bantam chickens. (A) The lowest error was observed at K = 10. (B) At K = 2, the SBxx population was first separated. At K = 3, the BHxx and ORge populations was separated. At K = 4, the COsch and ABwa populations were separated. At K = 6, the LSxx population was separated. At K=7, the SBxx population differentiated into two subpopulations. At K = 8, the FZxx population also divided into two subpopulations. At K = 9, the BHxx population further subdivided into two subpopulations. It is noteworthy that at K = 10, At K = 10, GBxx remains undifferentiated and exhibits a mixed ancestry from several breeds, even at K = 15, GBxx still does not show distinct differentiation. CV, cross-validation; K, The optimal population number; SBxx, Sebright Bantam; BHxx, Brahma; ORge, Orpington; COsch, Cochin; ABwa, Antwerp Bearded; LSxx, Langshan; FZxx, Booted Bantam; GBxx, Barbue du Grubbe.

Through Admixture analysis (Figure 2), the optimal fit curve for population clustering was calculated, revealing that the lowest error was observed at K = 10. At K = 2, the SBxx population was first separated. At K = 3, the BHxx and ORge populations was separated. At K = 4, the COsch and ABwa populations were separated. At K = 6, the LSxx population was separated. At K = 7, the SBxx population differentiated into two subpopulations. At K = 8, the FZxx population also divided into two subpopulations. At K = 9, the BHxx population further subdivided into two subpopulations. It is noteworthy that at K = 10, At K = 10, GBxx remains undifferentiated and exhibits a mixed ancestry from several breeds, even at K = 15, GBxx still does not show distinct differentiation. Understanding the relationship of population structure is highly significant for determining population differentiation, carrying out genetic improvement of varieties and selective breeding.

### Runs of homozygosity islands analysis

**Figure 3:**
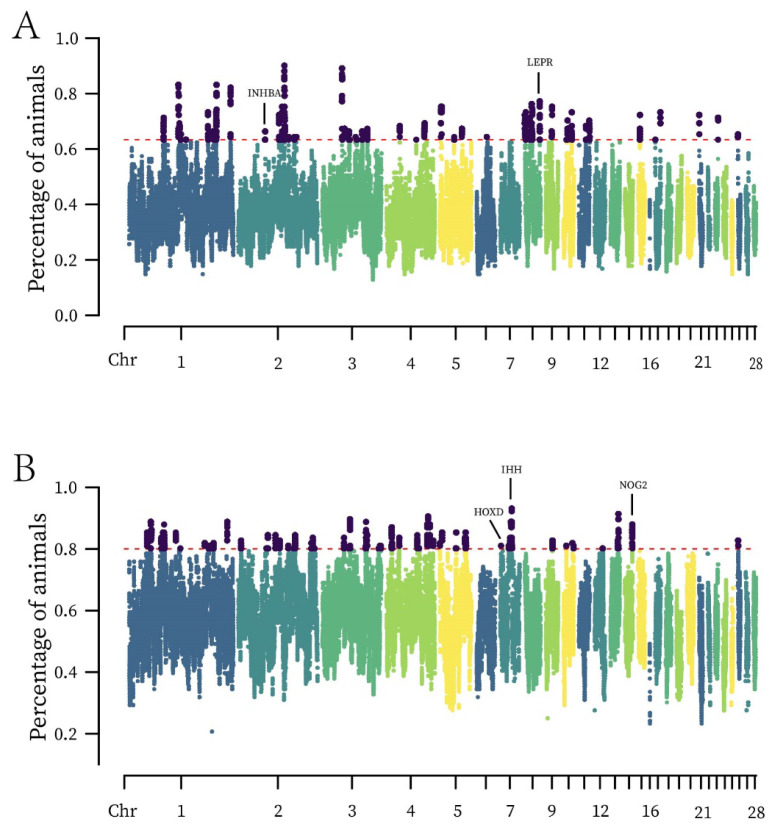
ROH islands across the entire genome of large and bantam chickens. The red dashed line represents the set threshold line, and the areas above the red dashed line represent the loci under selection. (A) *INHBA*, *LEPR*, and other genes have been selected for in large chickens. (B) *IHH*, *NOG2*, *HOXD* family, and other genes have been selected for in bantam chickens. Chr, chromosome; ROH, runs of homozygosity.

The study examined the distribution of ROH islands in populations of large and bantam chickens and visualized them based on SNP positions on chromosomes (Figure 3). In large chickens, 48 ROH islands were identified; in bantam chickens, 56 ROH islands were identified. We focused on the selected ROH islands for analysis and identified 2 genes related to skeletal development and weight regulation in large chickens, and 6 genes in bantam chickens (Table 2). Interestingly, we observed overlapping regions between the identified ROH island regions on chromosome 2 and chromosome 8 in large chickens, which coincided with the detected selection signatures and encompassed the *INHBA* and *LEPR*. Similarly, there was an overlapping region on chromosome 14 in bantam chickens, annotated to the *NOG2*. Analysis of ROH islands can help us understand the selected regions and genes of chickens with different body sizes under long-term natural and artificial selection.

### Selection signatures analysis

**Figure 4:**
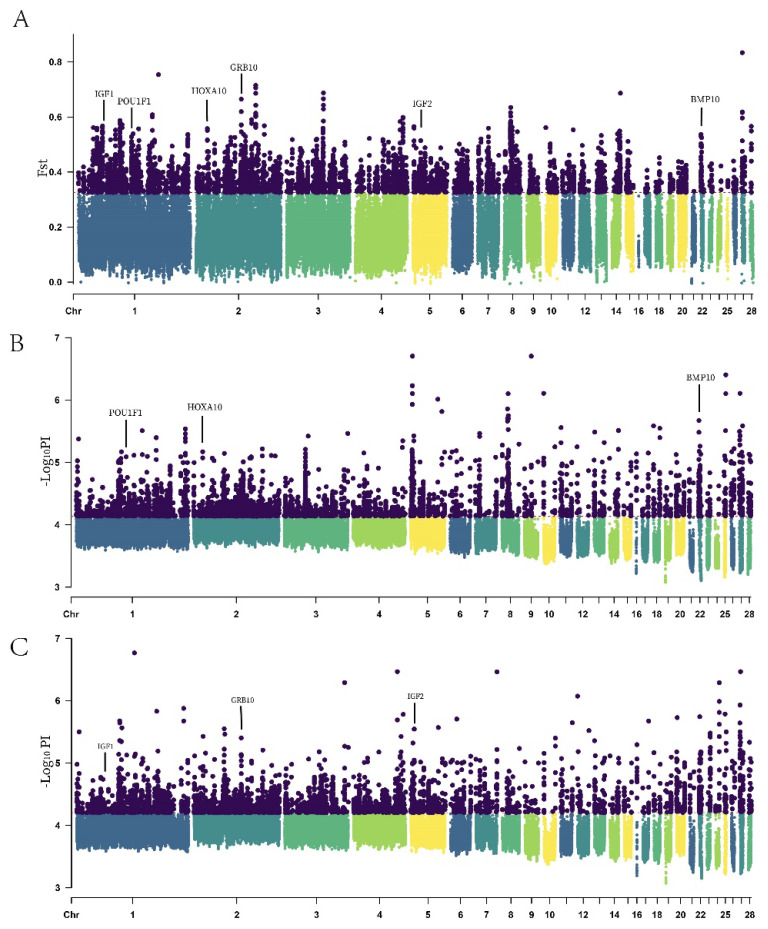
Manhattan plots of FST and π for large and bantam chickens. The red dashed line represents the set threshold line, and the areas above the red dashed line represent the loci under selection. (A) The distribution of the locations of genes such as *HOXA10, POU1F1, BMP10, IGF1, IGF2, GRB10* on the chromosomes. (B) *HOXA10, POU1F1* and *BMP10* have been selected in large chickens. (C) *IGF1, IGF2* and *GRB10* have been selected in bantam chickens. FST, population differentiation index; π, nucleic acid diversity.

**Figure 5:**
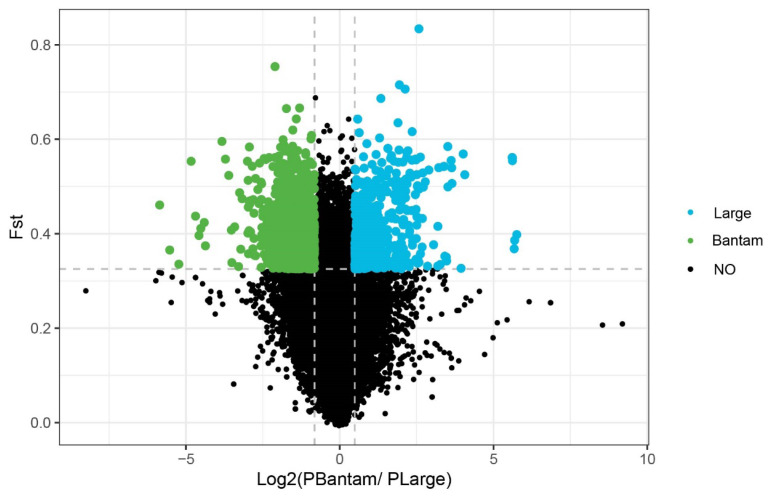
Visualization of π ratio for large and bantam chickens. Blue indicates positive selection in large chickens, while green indicates positive selection in bantam chickens. Fst, population differentiation index; π, nucleic acid diversity.

To identify candidate genomic regions under selection during the growth and development processes in large and bantam chicken populations, this study utilized the Fst and π radio methods to detect selection signatures. The top 5% threshold was employed as the selection criterion. If a region was significantly detected by both methods, it was considered a genuine selected region. (Figure 4) illustrates the Manhattan plots for different chicken body sizes, generated using the Fst and π methods, with the top 5% threshold serving as the selection criterion. (Figure 5) depicts the selected regions in different chicken body sizes using the Fst-π radio method. In large chicken population, 701 selected regions were identified, while in bantam chicken population, 1115 selected regions were detected.

**Figure 6:**
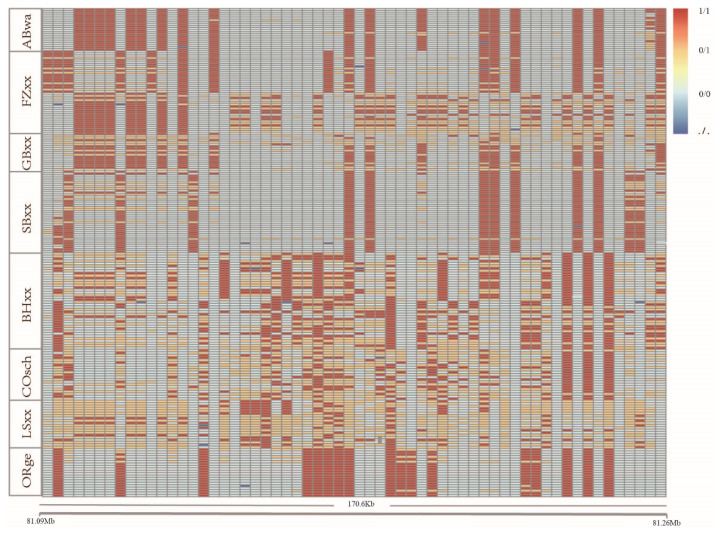
Haplotype analysis of *GRB10* in the region of chromosome 2 (81099018–81269619) for large and bantam chickens. Different changes in color represent changes in different genotypes. ABwa, Antwerp Bearded; FZxx, Booted Bantam; GBxx, Barbue du Grubbe; SBxx, Sebright Bantam; BHxx, Brahma; COsch, Cochin; LSxx, Langshan; ORge, Orpington.

The region of chromosome 2 (81099018–81269619) in bantam chickens, specifically within the *GRB10*, exhibits a strong selection signature. Haplotype analysis was conducted on this region, and the results are depicted (Figure 6). In this region, there are differences in genotypes at various loci between large and bantam chickens.

### Functional annotation and enrichment analysis

**Figure 7:**
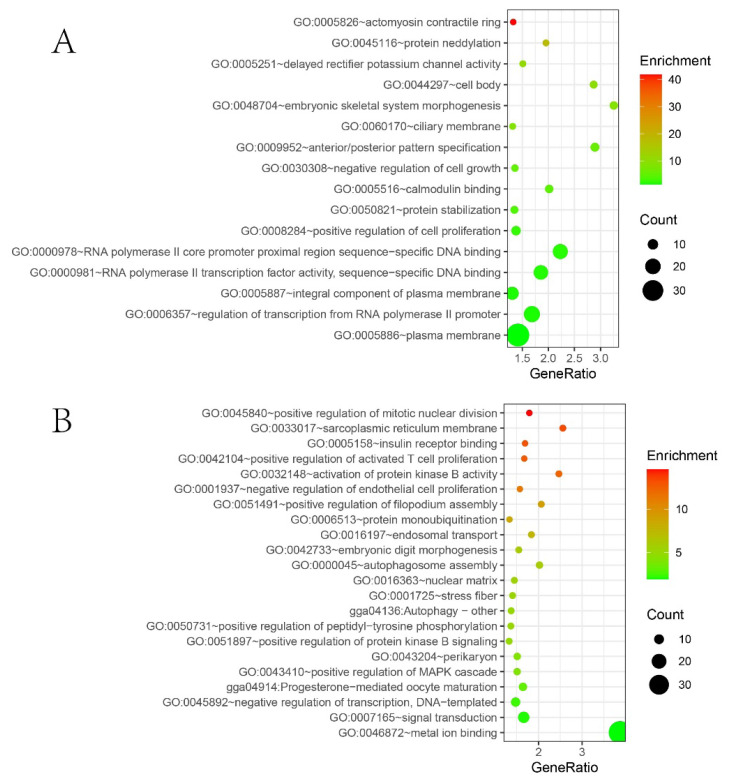
Bubble plot illustrating the GO and KEGG enrichment results for selected genes. The size of count represents the number of genes enriched in the pathway, and the change in color represents the fold enrichment of that pathway in the target gene set compared to the entire reference gene set. (A) Bubble plot illustrating the GO and KEGG enrichment results for selected genes in large chickens. (B) Bubble plot illustrating the GO and KEGG enrichment results for selected genes in bantam chickens. GO, gene ontology; KEGG, Kyoto encyclopedia of genes and genomes.

To understand the gene and functional differences in the selected regions between large and bantam chickens, this study annotated the 701 selected regions detected in the large chicken population, annotations for 322 genes. Similarly, the 1115 selected regions detected in the bantam chicken population were annotated, with annotations for 447 genes. The annotated genes were subjected to GO functional enrichment analysis and KEGG pathway analysis to gain insights into their functional features. The results of the enrichment analysis are presented in (Figure 7). In the large chicken population, a total of 16 enriched GO terms were identified. These terms are primarily associated with embryonic skeletal system morphogenesis, negative regulation of cell growth, calcium ion binding, and positive regulation of cell proliferation. In the bantam chicken population, a total of 20 enriched GO terms and 2 enriched KEGG pathways were obtained. The annotated GO terms are mainly related to insulin receptor binding, positive regulation of mitotic nuclear division, positive regulation of activated T cell proliferation, and positive regulation of the mitogen-activated protein kinase (MAPK) pathway. The annotated KEGG pathways are associated with autophagy and progesterone-mediated oocyte maturation. By selection signatures analysis and enrichment analysis, genes related to chicken body size and growth and development were identified, which laid a foundation for the subsequent development of molecular markers.

## DISCUSSION

Chickens are one of the most extensively studied livestock and hold significant importance in human production and daily life. Body size serves as a crucial economic trait, displaying considerable variations among various domesticated animals, including dogs, pigs, and chickens. Not only is body size a significant commercial characteristic in food production, but it is also a focal point in evolutionary and developmental biology research [2,7,19,20]. Moreover, body size is a typical feature of many complex traits, and it is widely considered to be influenced by numerous genes involved in similar functional pathways [21].

### Population structure

The results of population structure analysis show that large chickens cluster together prominently and exhibit close genetic distance, which is primarily associated with their origins and population history [22]. Bantam chickens exhibit relatively more dispersion, however, GBxx and ABwa cluster together and occupy the same branch on the evolutionary tree, which is consistent with a findings reported [23]. In the analysis of evolutionary tree and ancestral composition, SBxx, FZxx, and BHxx are all divided into two subpopulations. This is in line with the fact that each of SBxx (golden feathers and silver feathers), FZxx (white feathers and black feathers), and BHxx (light-colored feathers and dark-colored feathers) has two feather color phenotypes [24]. Population separation happens due to the fact that different populations possess distinct genotypes at the same locus. These different genotypes symbolize different genetic information and have diverse gene functions and roles, thereby giving rise to differences at both the phenotypic and genetic levels. It is worth noting that in the admixture analysis, The GBxx bloodline is relatively complex and has been involved in genetic flow events with other breeds, which is consistent with its use and history [23].

### Runs of homozygosity islands

In this study, we focused on specific genomic regions associated with chicken skeletal development and weight regulation, identifying a total of 8 relevant genes. Among them, the *INHBA, LEPR*, and *NOG2* overlapped with the genes identified by selection signatures. *INHBA*, located on chromosome 2 in large chickens, is potentially involved in the hedgehog signaling pathway, which plays a crucial role in the formation of growth plates in long bones [25]. *LEPR*, was discovered on chromosome 8 in large chickens and is important for weight regulation. In bantam chickens, we found the HOXD family (*HOXD3, HOXD4, HOXD8, HOXD9*) on chromosome 7, which has a significant impact on vertebrate axial skeletal development [26]. *IHH* is also located on chromosome 7 in bantam chickens and collaborates with parathyroid hormone-related protein to regulate the development of growth plates and long bones [27]. Additionally, during early cartilage development, *IHH* is a major source of the hedgehog signaling pathway, exhibiting proliferative expression in limb buds and eventually differentiating into skeletal chondrocytes. Mutations in the *IHH* can lead to defective cartilage development, affecting the proliferation of cartilaginous tissue and bone formation [25]. *NOG2*, located on chromosome 14 in bantam chickens, is a bone morphogenetic protein (BMP) antagonist. Currently, its function and mechanism of action in chickens are not well understood. We suppose that it may interact with BMPs in chicken skeletal development, regulating the proliferation and differentiation of bone cells, thereby influencing normal bone development in chickens.

### Selection signatures

The genomic regions under selection were determined by selecting the top 5% Fst and π ratio and taking their intersection. This approach combines Fst and π ratio, provides more robust and reliable results [28]. The candidate genes identified were further investigated using GO and KEGG enrichment analysis. Notably, several genes associated with chicken body size and growth were discovered in the analysis conducted on large chickens. *HOXA 10* is a member of the homeobox gene family, with a high degree of conservation. While the current understanding of *HOXA10* function primarily focuses on embryonic development, there is research indicating its vital role in adult skeletal formation and bone healing [29]. In large chickens, this gene has been positively selected, suggesting it may impact on skeletal formation and development influence chicken body size. A study revealed that the allele *B* of the *POU1F1* gene is likely to positively affect the growth traits on Nanyang cattle [30]. Similarly, *POU1F1* in native Korean chicken breeds showed that *POU1F1* polymorphism significantly influenced the growth and growth curve characteristics of chickens [31]. This shows that the *POU1F1* gene has a great relationship with the weight and growth and development of the animals. Overexpression of *BMP10* in mice has result in excessive myocardial growth and the formation of hyper trabeculation during embryonic development [32]. Transgenic mice with myocardial overexpression of *BMP10* show a significant reduction of 50% in heart size, as well as decreased body weight and size at one month of age; in a manner similar to mice, overexpression of *BMP10* in zebrafish leads to shortened body length, this implies that *BMP10* plays a crucial role in determining body size in vertebrates [33]. There are reports suggesting that *BMP10* also plays a role in inducing cell apoptosis, proliferation and growth [34].

Insulin-like growth factor I (*IGF1*) is a selected gene in bantam chickens and is known to affect body size in mice and humans [35]. Additionally, previous report has demonstrated that *IGF1* allele is a major determining factor for smaller body size in dogs [2], which is consistent with the findings of this study. Other studies have indicated that *IGF1* promotes longitudinal bone growth through its “insulin-like” metabolic effects, leading to the enlargement of chondrocytes [36], thereby influencing growth, development, and changes in body size. The synthesis and metabolic functions of growth hormone are primarily mediated by *IGF1*, suggesting that *IGF1* is the main determinant of cellular growth [37]. Rare mutations in the human *IGF1* result in severe growth inhibition and intellectual disability [38]. Mice with a knockout of the *IGF1* were born with only 60% of normal birth weight, and a few of the surviving adult mice even exhibit a body size less than one-third of that of normal mice [39], this indicates that *IGF1* in animal body size development. Insulin-like growth factor II (*IGF2*) is a crucial growth factor in embryonic and placental development, and its deficiency leads to severe growth retardation. *IGF2* primarily functions through the *IGF1* receptor in embryos and an unidentified receptor in the placenta [40]. Additionally, *IGF2* is indispensable for bone development as it stimulates the proliferation and differentiation of bone cells. The down regulation of *IGF2* is likely the underlying cause of the reduced bone mass observed during cortisol treatment [41]. The growth of developing mouse embryos is predominantly controlled by *IGF2* [42]. Moreover, *IGF2* was the first identified imprinted gene, and when targeted *IGF2* deletion is paternally transmitted, mouse embryos inherit only the inactive maternal allele, resulting in impaired development at birth [43]. Conversely, overexpression of *IGF2*, achieved through disruption of inhibitory *IGF2*R [44]or transactivation of *IGF2* [45], the fetus will display excessive growth. This suggests that *IGF2* can directly or indirectly influence changes in body size by affecting both bone development and embryonic growth. *GRB10* encodes a growth factor receptor-binding protein called Growth Factor Receptor-Binding Protein 10. This protein plays a crucial role in interacting with insulin receptors and insulin-like growth factor receptors, such as *IGF1*R and *IGF2*R. Overexpression of certain isoforms of *GRB10* can lead to the inhibition of tyrosine kinase activity, ultimately resulting in growth suppression. *GRB10* is also classified as an imprinted gene, in mice, the inactivation of the paternal allele of *GRB10* results in mice with enhanced aggression. When the maternal allele is inactive, the mice exhibit fetal overgrowth and are noticeably larger than their wild-type littermates, this demonstrates the gene’s role as a growth suppressor [46,47]. The gene may potentially influence changes in body size by affecting it through the inhibition of growth.

Finally, we have identified several genes associated with chicken body size as well as growth and development (Table 3). This offers a foundation for the functional analysis of relevant genes, the development of molecular markers, and the breeding of new varieties. This study also has some limitations. This analysis is carried out using natural populations, and it is impossible to ensure that the inherent genetic characteristics of different varieties will not interfere. However, when selecting and grouping varieties, we have taken this into account to ensure that it will not affect the analysis results. In general, this study will serve as a reference for analyzing the genetic mechanism of chicken body size. In future research, relevant causal mutation sites will be further identified to develop molecular markers for chicken body size and drive the development and progress of molecular breeding.

## CONCLUSION

The research results show that large chickens and bantam chickens are separated in population structure. The genetic distance within large chickens is closer, while the genetic distance within bantam chickens is farther. This is consistent with their historical origins and uses. By analyzing population structure, we can gain an understanding of the population divergence and genetic disparities among different groups. This is important for researching the evolution and adaptability of species and carrying out genetic improvement.

Among the ROH islands of interest, a total of 8 candidate genes were identified, primarily exerting influence on chicken size and growth by affecting skeletal development and weight regulation. Furthermore, in selection signatures analysis of chickens of different sizes, genes related to chicken body size, growth, and development were identified (*HOXA10*, *POU1F1*, *BMP10*, *IGF1*, *IGF2*, *GRB10*, etc.). Among them, *HOXA10*, *POU1F1* and *BMP10* are under positive selection in large chickens, which are mainly related to the body size, growth and development and bone formation of chickens. *IGF1*, *IGF2* and *GRB10* are under strong positive selection in bantam chickens, which mainly affect the body size of chickens by affecting bone growth, promoting or inhibiting embryonic development, etc. In this research, through analyzing the population structure, ROH islands, and selection signatures of different chicken body sizes (as a natural population is utilized, there will be inherent genetic characteristics), we have identified multiple genes associated with chicken body size and growth and development (such as *BMP10*, *IGF1*, *GRB10*, etc.). This offers a reference for developing molecular markers for body size, advancing the development and progress of molecular breeding, and analyzing the genetic basis of complex economic traits like chicken body size and growth.

## Figures and Tables

**Table 1:** Sample variety information summary table

Group	Large chickens				Bantam chickens			
Breed	Brahma	Cochin	Langshan	Orpington	Antwerp Bearded	Booted Bantam	Barbue du Grubbe	Sebright Bantam
Abbreviation	BHxx	COsch	LSxx	ORge	ABwa	FZxx	GBxx	SBxx
Subpopulation	2	1	1	1	1	2	1	2
Count	40	21	20	20	20	39	18	39

**Table 2:** List of candidate genes located in genomic regions with ROH islands in large and bantam chickens

Group	CHR	Start (bp)	End (bp)	Gene name
Large chickens	2	50745139	50762744	Inhibin beta A subunit (*INHBA*)
Large chickens	8	28434800	28465001	Leptin receptor (*LEPR*)
Bantam chickens	7	16325200	16354477	Homeobox D3 (*HOXD3*)
Bantam chickens	7	16340652	16342299	Homeobox D4 (*HOXD4*)
Bantam chickens	7	16355644	16357729	Homeobox D8 (*HOXD8*)
Bantam chickens	7	16361241	16364705	Homeobox D9 (*HOXD9*)
Bantam chickens	7	22427441	22435414	Indian hedgehog (*IHH*)
Bantam chickens	14	14084509	14085132	Noggin 2 (*NOG2*)

CHR, chromosome; ROH, runs of homozygosity; bp, base pair.

**Table 3:** List of selected genes in selection signatures of large and bantam chickens

Group	CHR	Start (bp)	End (bp)	Gene name	Number of variant SNPs
Large	1	94257934	94273544	*POU1F1*	13
Large	2	32651349	32654714	*HOXA10*	3
Large	22	421956	427810	*BMP10*	1
Bantam	1	55281097	55330373	*IGF1*	11
Bantam	2	80852532	80991947	*GRB10*	63
Bantam	5	13971786	13980128	*IGF2*	3

CHR, chromosome; bp, base pair.
